# A reassessment of spinal cord pathology in severe infantile spinal muscular atrophy

**DOI:** 10.1111/nan.13013

**Published:** 2024-10

**Authors:** Hazel Allardyce, Benjamin D. Lawrence, Thomas O. Crawford, Charlotte J. Sumner, Simon H. Parson

**Affiliations:** 1 Institute of Medical Sciences, School of Medicine, Medical Sciences and Nutrition, University of Aberdeen, Aberdeen, Scotland, UK; 2 Department of Neurology and Pediatrics, Johns Hopkins University School of Medicine, Baltimore, MD, USA; 3 Department of Neurology, Neuroscience, and Genetic Medicine, Johns Hopkins University School of Medicine, Baltimore, MD, USA

**Keywords:** chromatolysis, grey matter, morphology, motor, motor neuron disease, sensory, stereology, white matter

## Abstract

**Aims:**

Spinal muscular atrophy (SMA) is a life-limiting paediatric motor neuron disease characterised by lower motor neuron loss, skeletal muscle atrophy and respiratory failure, if untreated. Revolutionary treatments now extend patient survival. However, a limited understanding of the foundational neuropathology challenges the evaluation of therapeutic success. As opportunities to study treatment-naïve tissue decrease, we have characterised spinal cord pathology in severe infantile SMA using gold-standard techniques, providing a baseline to measure treatment success and therapeutic limitations.

**Methods:**

Detailed histological analysis, stereology and transmission electron microscopy were applied to post-mortem spinal cord from severe infantile SMA patients to estimate neuron number at the end of life; characterise the morphology of ventral horn, lateral horn and Clarke’s column neuron populations; assess cross-sectional spinal cord area; and observe myelinated white matter tracts in the clinically relevant thoracic spinal cord.

**Results:**

Ventral horn neuron loss was substantial in all patients, even the youngest cases. The remaining ventral horn neurons were small with abnormal, occasionally chromatolytic morphology, indicating cellular damage. In addition to ventral horn pathology, Clarke’s column sensory–associated neurons displayed morphological features of cellular injury, in contrast to the preserved sympathetic lateral horn neurons. Cellular changes were associated with aberrant development of grey and white matter structures that affected the overall dimensions of the spinal cord.

**Conclusions:**

We provide robust quantification of the neuronal deficit found at the end of life in SMA spinal cord. We question long-accepted dogmas of SMA pathogenesis and shed new light on SMA neuropathology out with the ventral horn, which must be considered in future therapeutic design.

## INTRODUCTION

Severe infantile spinal muscular atrophy (SMA) is a debilitating childhood motor neuron disease, which without medical intervention is commonly fatal in infancy [1]. SMA is caused by pathogenic variants in the survival motor neuron 1 (*SMN1*) gene, resulting in reduced expression of the ubiquitous and essential survival motor neuron (SMN) protein. In the absence of *SMN1*, ‘backup’ *SMN2* genes provide sufficient functional protein for short-term survival but insufficient to prevent motor neuron degeneration, resulting in progressive muscle atrophy, weakness and death [2].

Degeneration and specific loss of alpha motor neurons, central chromatolysis of remaining motor neurons and mis-migrated motor neurons are described as ‘characteristic’ of SMA pathology [3–6]. However, this dogmatic view of SMA is based on scant original data, small patient cohorts and inadequate quantitative techniques. Recent work has demonstrated a far wider range of affected cells and tissues in SMA, in both patients and disease models [7, 8]. Even within the nervous system, SMA pathology additionally manifests in astrocytes, microglia, oligodendrocytes and blood vessels [9–14].

The few studies that have assessed motor neuron loss in SMA spinal cords used inadequate quantitative techniques without consideration of spinal level, resulting in a reported range of neuron loss between 50% and 95% [29, 48, 54, 55, 60]. Stereology is the gold-standard technique for quantification of three-dimensional structures from the observations of two-dimensional sections [15], but only one study has applied this methodology showing an approximate 73% loss of motor neurons at an unspecified spinal level, which may not be present throughout the whole cord [16]. It is essential to understand the degree of loss, as current therapies can only rescue surviving neurons.

All previous studies have focussed on the cervical and lumbar expansions of the spinal cord which supply the upper and lower limbs, respectively. Although the phenic nerve (cervical 3–5), and therefore the innervation of the diaphragm, is classically spared in SMA [17, 18], respiratory failure due to loss of thoracic motor neurons and denervation of the intercostal muscles is the leading cause of fatality in SMA Type I and II patients [19–21]. Thoracic motor neurons also innervate the paraspinal muscles which control posture and prevent scoliosis, a common co-morbidity in severe SMA patients, which negatively affects both respiratory and motor function [22–24]. Clinicians recognise a key early feature of severe infantile SMA is the inability to maintain a straight trunk when the pelvis or shoulder girdle is turned (loss of “logrolling”), largely driven by trunk proprioceptive input and efferent discharge of intercostal and superficial abdominal muscles [25]. Thoracic spinal motor neurons are affected early in the disease, and therapeutic delay may be clinically relevant [26]. The thoracic spinal cord also provides a window into the effects of SMA on autonomic neurons and descending pathways and is therefore an ideal region to reassess spinal cord pathology in SMA.

Revolutionary treatments are now widely available, improving motor function, and extending the life expectancy of SMA patients [27]. These therapies upregulate SMN protein levels either by increasing full-length transcripts from *SMN2*, nusinersen (Spinraza; Biogen, Cambridge, MA) and risdiplam (Evrysdi; Roche, Basel, Switzerland), or by rescuing the *SMN1* gene, onasemnogene abeparvovec (Zolgensma, AveXis, Novartis, Chicago, IL). Each is designed to halt motor neuron loss and disease progression [27]. With treatments now widespread, the opportunity to collect foundational data from the few samples of untreated SMA spinal cord is fast diminishing.

Here, we use gold-standard stereological techniques to robustly characterise disease pathology in the thoracic spinal cord of a cohort of severe infantile SMA. We report substantial motor neuron loss within the ventral horn even in the youngest SMA cases, abnormal morphology of remaining motor neurons and early degenerative changes in neurons of the sensory associated Clarke’s column. In contrast, sympathetic lateral horn neurons were preserved. Gross abnormalities of spinal cord dimensions reflected aberrant development of grey matter and white matter. These data reassess baseline severe SMA neuropathology that will allow evaluation and understanding of current therapeutic success and should be considered in the design of novel ones.

## MATERIALS AND METHODS

### Sample collection

Short post-mortem interval, paediatric human spinal cord samples from SMA patients (*n* = 6) and similarly age-matched controls (*n* = 6) were obtained from brain banks: BRAIN UK, NIH NeuroBioBank and John Hopkins University Medical School. At Johns Hopkins University Medical School and the NIH NeuroBioBank, expedited autopsies were conducted under parental- or patient-informed consent in strict observance of the legal and institutional ethical regulations, and material transfer agreements were obtained between collaborating institutes. Tissue samples obtained were dissected at autopsy, fixed in 4% paraformaldehyde (room temperature)/2% glutaraldehyde (4°C) for 24 h, washed and stored in PBS. Samples were transferred to the University of Aberdeen as fixed tissue blocks, where they were cryopreserved and transversely serially cryosectioned (8 μm) for downstream analysis. From BRAIN UK, samples were consented for research, dissected at autopsy, fixed in formalin, embedded in paraffin wax and transferred to the University of Aberdeen as FFPE blocks, where they were transversely serially sectioned (8 μm) and stained for downstream analysis.

### Stereological neuron quantification

Stereological methods were applied for ventral horn neuron quantification. To follow the physical disector technique, spinal cord samples were serially sectioned and consecutive section pairs were analysed. Systematic uniform random sampling was implemented using a random number generator to determine the first section in the series. From here, every 12th section (the ‘reference’) and the section immediately following (the ‘look-up’) were selected for analysis. Section thickness, also known as disector height, was 8 μm, and the sampling fraction was 1/12th, selected based on the literature regarding neuron soma size. The sections were stained with the Klüver-Barrera method, 0.1% Luxol fast blue, 56°C (6 h) and 0.1% cresyl violet, 37°C (4 min) to visualise neurons and demarcate grey from white matter. Sections were imaged on a Zeiss AxioScan Z1 and viewed using the Zen software. Comparing section pairs, newly appearing neurons with visible nuclei were counted in the entire ventral horn in a total of 10 section pairs per cord. The criteria for a neuron were a large cell soma containing Nissl bodies located in the ventral horn, with a visible nucleus only on the ‘reference’ section.

The ventral horn area was quantified by measuring the traced area on ImageJ, and the Cavalieri method was applied to estimate volume [28]. The volume fraction of the ventral horn to the whole spinal cord in a cross-section was calculated as a percentage. Neuronal density (per mm^3^ of ventral horn) was estimated using the equation *Nv* = *Q*^−^/(*a* * *t*), where *Q*^−^ is the sum of neurons counted, *a* is the sum of ventral horn areas sampled, and *t* is the section thickness. The absolute number of ventral horn neurons was estimated using the equation *A* = *Nv* * (*a* * *sf* * *t*), where *Nv* is the neuronal density, *a* is the sum of the ventral horn area sampled, *sf* is the reciprocal of the sampling fraction and *t* is the section thickness. These estimates were made in the ventral horn of a 960-μm length of the thoracic spinal cord, as we did not have access to the entire spinal cord, and were scaled and expressed as an absolute number of ventral horn neurons in a 1-mm length of thoracic spinal cord. Due to the limited area of the ventral horn, counting frames were not required and the entire ventral horn area was assessed. The coefficient of error was calculated using the equation $CoE\;\left(\%\right)=\left(\surd [k/k-1\{\left(\sum Q^{-2}/\sum Q^{-}\sum Q^{-}\right)+\left(\sum a^{2}/\sum a\sum a\right)+\left(\sum Q^{-}a^{2}/\sum Q^{-}\sum a\right)\right)*100$, where *k* is the number of section pairs analysed, *Q*^−^ is the sum of neurons counted and *a* is the sum of ventral horn areas sampled. *CoE* should be equal to, or below, 15% for control tissues [29]. The coefficient of variation ([StDev/Mean] * 100) was also calculated. *N* = 6 control and *N*=*6* SMA spinal cord samples for estimation of ventral horn neuron density and absolute number.

It was not possible to determine a stereological estimation of the lateral horn neuron density as lateral horn volume could not be measured due to the lack of features to identify regional margins. Here, the physical disector principle was applied to estimate the lateral horn neuron number, which was then multiplied by the reciprocal of the sampling fraction. This count is an estimate of the number of lateral horn neurons in a 1-mm length of the thoracic spinal cord. *N* = 3 control and *N*=*3* SMA spinal cord samples for estimation of lateral horn neuron number.

### Ventral horn neuron size

Maximum cell soma diameter was measured through a central profile of each neuron with a visible nucleus, using ImageJ to calculate the maximum calliper diameter. Results presented as median (interquartile range). A histogram comparing the frequency of ventral horn neuronal diameters between control and SMA cases is shown with a log-normal distribution fit, indicated as the best fit using individual distribution identification.

### Neuron morphology

SMA ventral, lateral and Clarke’s column neurons were categorised as having normal or abnormal morphology. Normal morphology was assigned by the presence of a large, multipolar cell soma, a centrally placed nucleus and dark purple Nissl staining. Abnormal morphology was assigned by either (1) a small and rounded cell body, (2) an eccentrically displaced nucleus or (3) changes in Nissl substance distribution.

### Cross-sectional area analysis

Four Sudan black stained sections (0.5% Sudan black in 70% ethanol [5 min] followed by washes in dH_2_O and 0.1% PBS) were imaged (Zeiss AxioScan Z1), and area measurements of entire cord cross-sections, total grey matter and white matter, and area of four regions of interest (ROIs): ventral horn, dorsal horn, lateral corticospinal tract and dorsal column were quantified by tracing the peripheral borders of each using the ImageJ. As no clear delineation exists between the lateral corticospinal tract and the posterior spinocerebellar tract, the entire area from the grey matter to the periphery was measured.

### EM and myelinated axons

Epoxy resin–embedded blocks for transmission electron microscopy (TEM) were prepared at Johns Hopkins University, control *N* = 2 and SMA *N* = 4, as described in [30]. Grids were prepared by the microscopy and histology facility, University of Aberdeen, using a diamond knife to cut ~90 nm sections onto formvar/carbon-coated copper grids and contrast stained with Uranyless and Lead citrate. Twenty micrographs of white matter tracts (excluding the ventral root exit zone) at magnification ×2500 were obtained from each sample in a random but systematic manner. One single image was taken per grid square, if the entire square contained only white matter, determined by the high density of myelinated axons present.

### Statistical analysis

Statistical analysis was calculated on GraphPad Prism software (V9) and Minitab (V21.4) and included descriptive statistics, removal of outliers (ROUT method, *Q* = 1%) and Shapiro–Wilk normality testing. Unpaired *T*-tests with Welch’s correction were applied to normally distributed data. Non-parametric Mann–Whitney tests were used to compare the distribution of non-Gaussian data. Pearson correlation coefficient tested the hypothesis of increasing neuron loss with increasing age. Bonett’s method assessed variance in neuron diameter distribution. Simple linear regression analyses show changes in the cross-sectional spinal cord area over time. Slope value (s) defines the steepness of the regression line, and therefore the change in *Y* for each unit change in *X* (*DY*/*X*), positive indicating incline (growth) and negative as decline (atrophy). *R*^2^ indicates the goodness of fit of a simple linear regression model. *p* value details whether the slope is significantly non-zero as calculated by the *F* test and its degrees of freedom, with *p* < 0.05 indicating a significant change over time, as shown on graphs. Linear regression analyses are reported as (slope value [s], *R*^2^, *F* [degrees of freedom] value, *p* value). In all subsequent statistical analysis, *p < 0.05, **p < 0.01, ***p < 0.001 and ****p < 0.0001 were taken as significant.

## RESULTS

We systematically assessed the macro- and micro-anatomy of the spinal cord in a cohort of severe infantile SMA individuals with survival times of 1 to 12 months. Patient demographics are presented in Table 1. All but one SMA case within this cohort, whose genotype remains unknown, possessed two copies of *SMN2*. The case with the short postnatal survival of just 7 days is likely to reflect either one or two copies of *SMN2*, as three copies of the *SMN2* gene are associated with significantly longer survival time [31–33].

### Motor neuron loss is substantial but variable at the end of life in SMA patients

Applying stereological methods, we quantified the mean neuronal density and absolute number of neurons in the ventral horn of 1 mm length of the thoracic spinal cord. An unbiased approach of assessing all neurons, alpha and gamma, was applied as there remains uncertainty in the ability to differentiate between these populations in human spinal cord, especially in the disease state.

**FIGURE 1:**
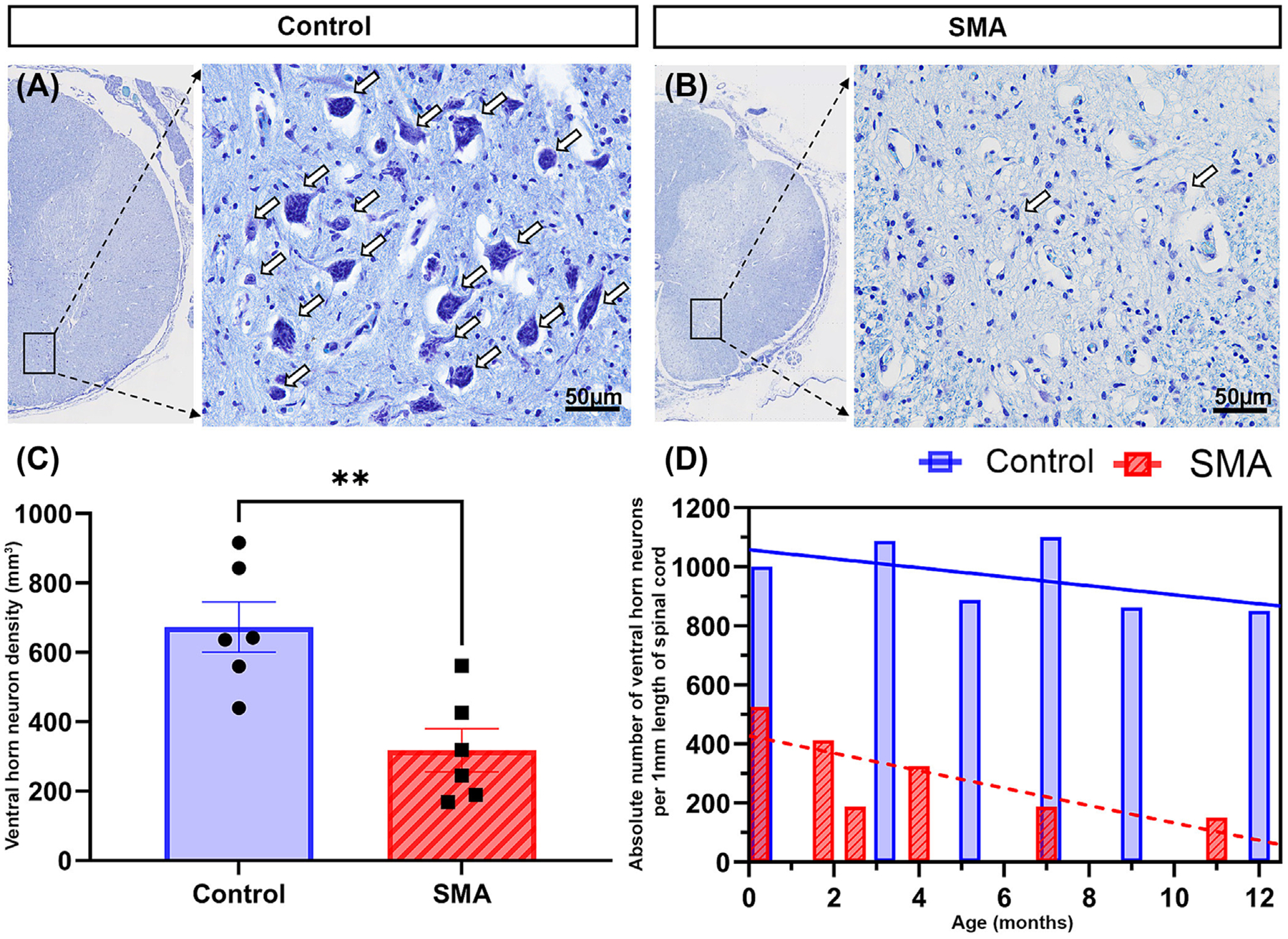
Representative micrographs of ventral horn neurons in control (A) and SMA (B) thoracic spinal cord. Arrows show ventral horn neuron cell soma. Scale bar, 50 μm. (C) Ventral horn neuron density in control (672.8 ± 72.53 neurons/mm^3^) and SMA (317.8 ± 62.10 neurons/mm^3^) in the thoracic spinal cord. The results are presented as mean ±SEM. *p* value calculated by unpaired *t*-test with Welch’s correction, **p < 0.0042. (D) The absolute number of ventral horn neurons in 1-mm length of the thoracic spinal cord, control range = 850–1100 neurons, and SMA = 150–525. Pearson correlation coefficient, control *r* = −0.56 (*p* = 0.25) and SMA *r* = −0.78 (*p* = 0.07). Data was collected from >400 tissue sections and observation of >3000 neurons. SMA, spinal muscular atrophy.

Quantification confirmed the initial observations (Figure 1(A,B)) that ventral horn neuronal density was significantly decreased in SMA spinal cords compared to controls (control = 672.8 ± 72.53 neurons/mm^3^ [mean ±SEM] CoE 10.93% ± 0.68, CV 26.4%; SMA = 317.8 ± 62.1 neurons/mm^3^, CoE 21.79% ± 1.95, CV 47.9%) **p < 0.0042 (Figure 1C). The SMA ventral horn was of a significantly smaller volume in the SMA spinal cord compared to control (control = 1.31 ± 0.11 mm^3^ [mean ±SEM]; SMA = 0.84 ± 0.09 mm^3^), **p = 0.0087. However, proportionally, it occupied the same volume of the spinal cord, control = 8.72 ± 0.63% (mean ± SEM), CoE 1.65 ± 0.16, CV 17.6% and SMA = 7.93 ± 0.78%, CoE 2.12 ± 0.43, CV 24.1% (Pns = 0.59).

The absolute number of ventral horn neurons per 1-mm length of the thoracic spinal cord in controls ranged between 850 and 1100 neurons (median = 944), while SMA thoracic spinal cord only contained between 150 and 525 neurons (median = 257), ****p < 0.0001. On average, 70% of the ventral horn neurons were lost at the end of life in severe infantile SMA patients (Figure 1D). Even in the youngest SMA case, the absolute number of neurons remaining was dramatically decreased (control = 1000 neurons, SMA = 525 neurons); this degree of loss generally increased with increasing age. Control cases showed a negative slope trend of absolute number of ventral horn neurons with increasing age (*r* = −0.56, *p* = 0.25), which likely reflects continuing postnatal growth and elongation of the spinal cord resulting in a gradual decrease in density rather than a change in the absolute number of neurons. SMA individuals showed a steeper trend rate of decline (*r* = −0.78, *p* = 0.07).

Clinically, all SMA cases showed extreme abnormalities in the achievement of motor milestones compared with non-SMA individuals (Table 2). The greatest progress towards motor milestones was generally achieved by the longer-surviving individuals; however, ventral horn number at the end of life was not a good measure of motor ability achieved during life. This is likely indicates the varied onset and progression of neuronal loss between individuals, as the maximum milestone will be achieved at an age when the number of functional motor units coincides with the age-appropriate ability to carry out specific motor functions.

### SMA ventral horn neurons are small, irrespective of survival time

**FIGURE 2:**
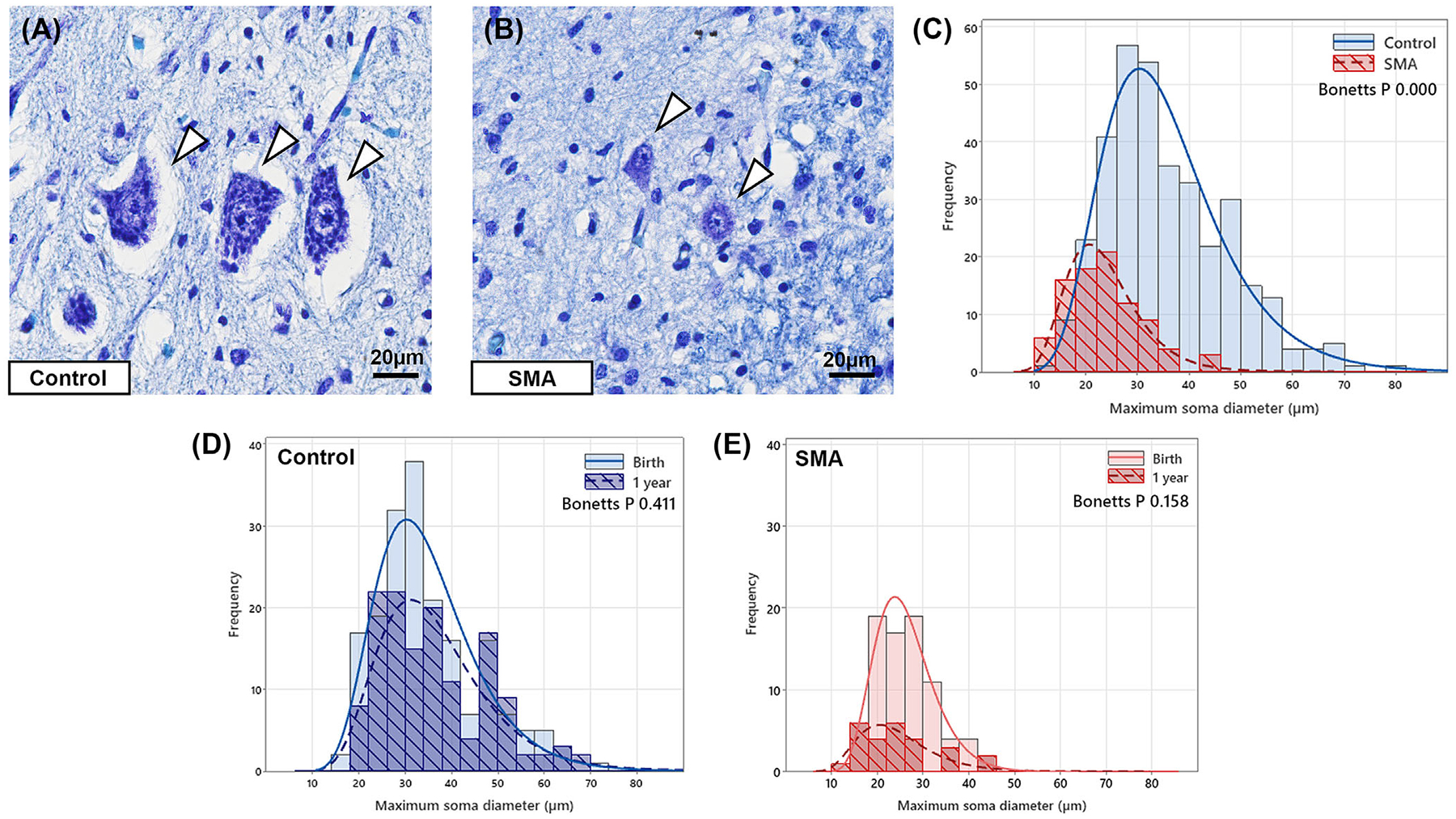
High-power representative micrographs of control (A) and SMA (B) motor neurons, scale 20 μm. Arrowheads show large motor neuron soma in controls, while SMA motor neurons are small. (C) Histogram comparing the pooled frequency of ventral horn neuronal diameters (μm) in all control (blue) and SMA (red striped) cases showing a distinct shift to the left in SMA cases, indicating small SMA neurons. Bonett’s test for comparing two population variances, ****P0.000. (D) Histogram comparing the frequency of ventral horn neuronal diameters (μm) in controls at birth (light blue) and at 1 year of age (dark blue striped) showing the maximum neuronal size is achieved by birth, Bonett’s test for comparing two population variances, Pns 0.411. (E) Histogram comparing the frequency of ventral horn neuronal diameters (μm) in SMA at birth (pink) and at 1 year of age (red striped) showing SMA motor neurons are always small irrespective of age at the endpoint. Bonett’s test for comparing two population variances, Pns 0.158. Lognormal distribution fit is shown on all histograms. Abbreviation: SMA, spinal muscular atrophy.

The remaining ventral horn neurons in SMA appeared noticeably smaller at all ages. Quantification of central profile soma size confirmed that control ventral horn neurons are large at birth (Figure 2(A)), while remaining SMA neurons were visibly and significantly smaller than those in aged-matched controls (Figure 2(B)); control = 33.6 μm (IQR 27.5–43.9); SMA = 22.5 μm (18.1–27.8), ****p <0.0001. Histogram of pooled ventral horn neuron diameters from all cases showed a distinct shift to the left (smaller neurons) in SMA (****p = 0.000: Figure 2(C)). SMA ventral horn contained no large (>46 μm diameter) neurons, fewer medium-sized neurons (18–46 μm), but an increased number of small neurons (10–18 μm). Notably, in controls, the maximum neuronal soma diameter was achieved by birth and remained unchanged up to 12 months, (Pns 0.0411: Figure 2(D)), while SMA ventral horn neurons were small at all endpoints (Pns 0.158: Figure 2(E)).

### Surviving ventral horn neurons display abnormal morphology in SMA

**FIGURE 3:**
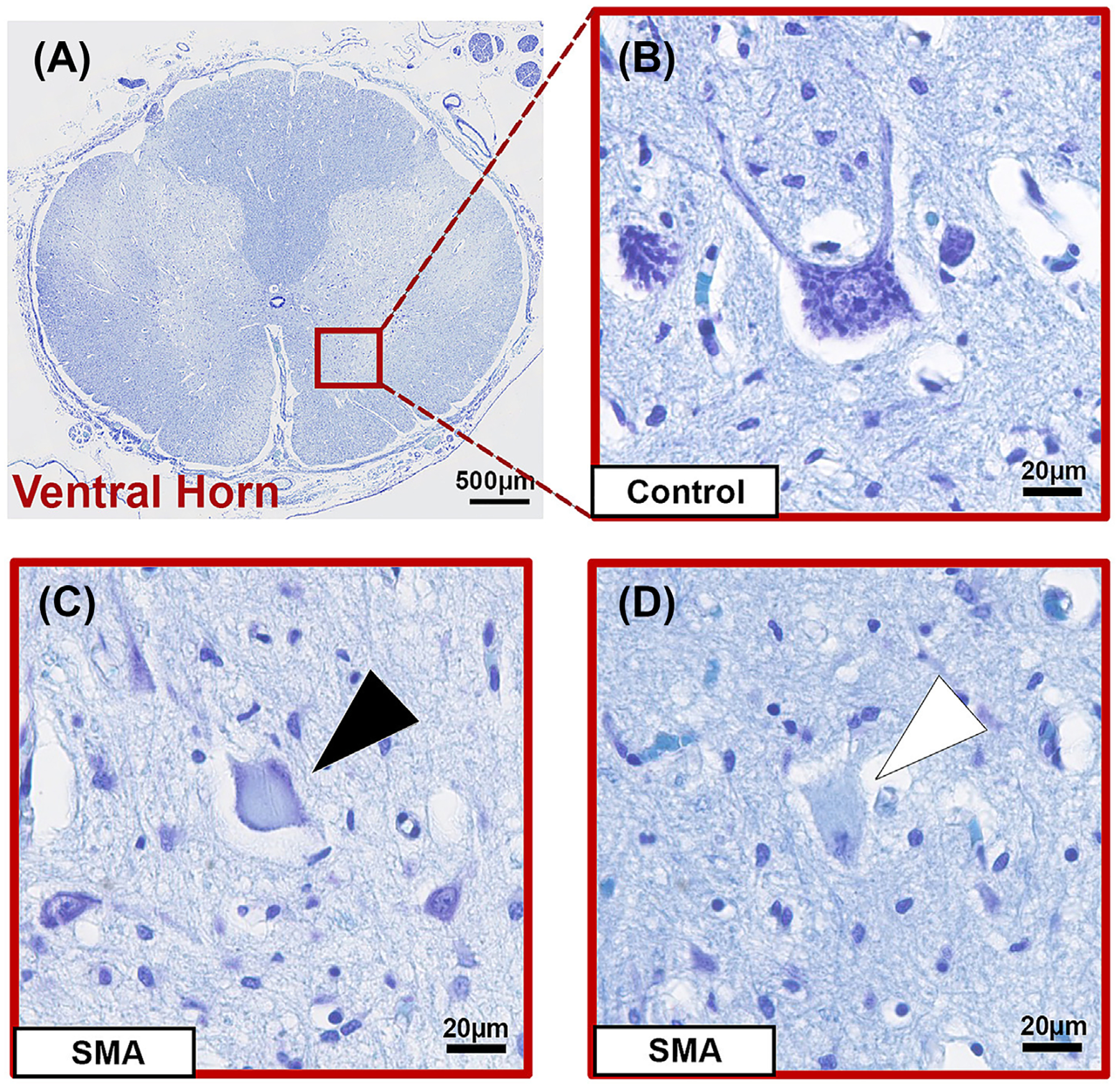
(A) Low-power micrograph showing the location of the ventral horn (red box) in the spinal cord, scale 500 μm. (b) High-power micrograph of healthy motor neuron in control spinal cord with large multipolar cell body, centrally located nucleus and clear nucleolus, and Nissl substance within the cytoplasm for reference, scale 20 μm. (C) Representative high-power micrograph of a motor neuron in SMA spinal cord with rounded cell body and peripherally displaced Nissl substance, indicated by a black arrowhead. (D) Representative high-power micrograph of a motor neuron in SMA spinal cord with no visible Nissl substance and disrupted nucleus, indicated by a white arrowhead. Scales 20 μm.

Normal, healthy motor neurons in control cases have a characteristic large multipolar cell body, with a centrally located nucleus, an abundance of Nissl substance throughout the cytoplasm, and projecting neurites (Figure 3(B)). Abnormal neurons in SMA cases were characterised by small and rounded cell bodies, redistribution or loss of Nissl substance and nuclear disruption. A lack of cresyl violet staining was observed in all SMA cases, indicative of a general loss of Nissl substance within the entire neuronal population. Key features of the stages of chromatolysis were also observed in some SMA neurons, including peripheralization of Nissl substance (Figure 3(C)) and eccentrically displaced nuclei. A proportion of remaining neurons contained no visible Nissl substance (achromasia) and showed nuclear disruption (Figure 3(D)). No swelling of neuronal cell bodies was observed. In contrast, some neurons were small with intense cytoplasmic staining and an uneven periphery, and projecting neurites were rarely observed in SMA neurons. We did not observe any mis-migrated motor neurons, located out with the ventral horn, but we were not able to assess ventral outflow and roots, where these have been reported previously. On average, 65% of remaining ventral horn neurons in SMA spinal cords were categorised as abnormal (range 27%–100%).

### Clarke’s column but not lateral horn neurons are damaged in SMA spinal cord

To determine if neuronal degeneration was limited to ventral horn motor neurons in SMA spinal cord, we assessed adjacent neuronal populations of Clarke’s column and the lateral (intermediate) horn of the thoracic spinal cord.

**FIGURE 4:**
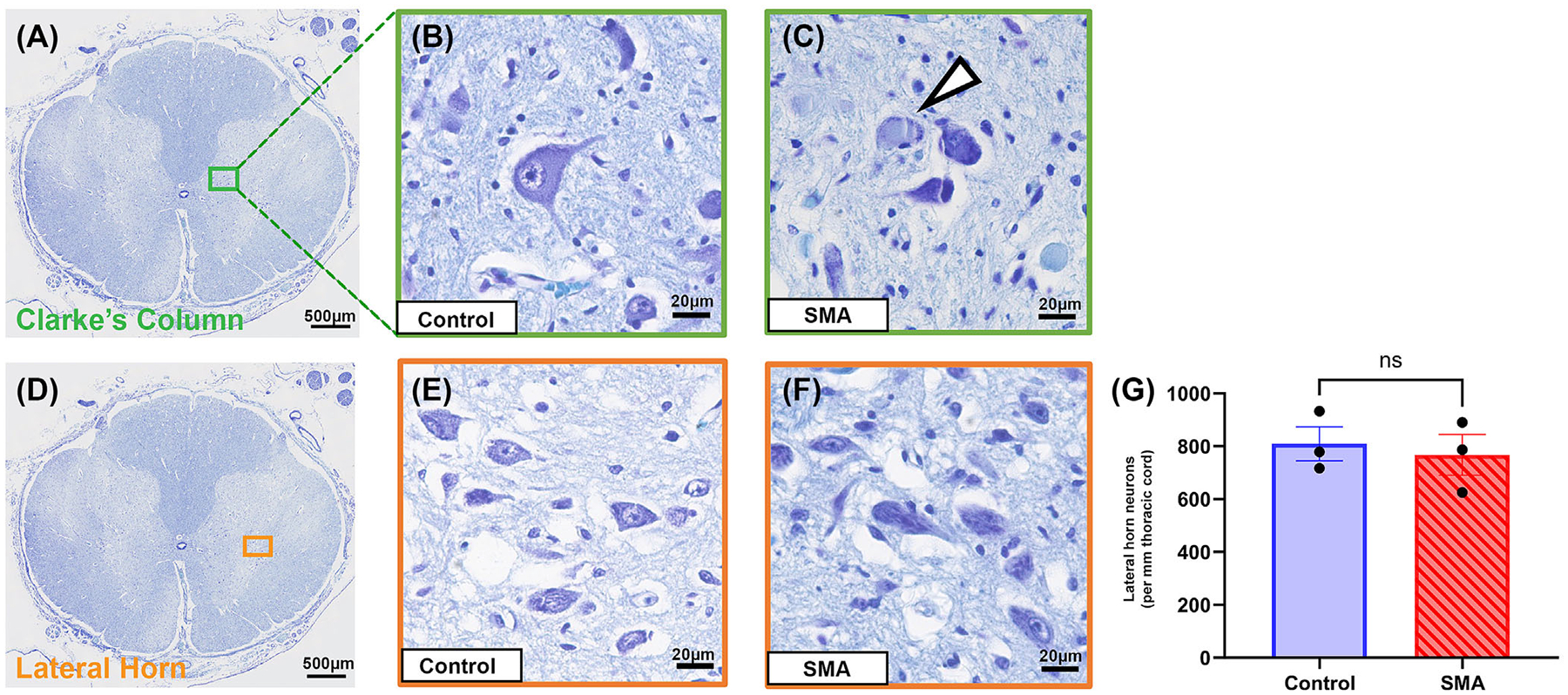
(A) Clarke’s column in the spinal cord, scale 500 μm. (B) A healthy Clarke’s column neuron in control spinal cord showing large cell soma with distinct nucleus, nucleolus and neurites. (C) Clarke’s column neurons with peripheral Nissl substance in SMA spinal cord, indicated by the arrowhead, scales 20 μm. (D) Lateral horn in the thoracic spinal cord, scale 500 μm. (E) Morphologically healthy lateral horn neurons in control and (F) in SMA spinal cord with multipolar cell body, centrally placed nucleus and nucleolus and cytoplasmic Nissl substance, scale 20 μm. (G) Lateral horn neuron number of control (809.6 ± 64.41) and SMA (767.5 ± 77.28) in the thoracic spinal cord, mean ±SEM per 1-mm length. *N* = 3. *p* value calculated by unpaired *t*-test with Welch’s correction Pns 0.70.

Neurons of Clarke’s nucleus are thought to be key to unconscious proprioceptive processing and appear oval or pyriform in shape with often peripherally located nuclei and homogenously distributed Nissl substance (Figure 4(A,B)). Clarke’s column was present in four SMA and four aged-matched control spinal cord samples. While Clarke’s column neurons of control cords displayed characteristic normal morphology, a portion of SMA neurons contained peripherally displaced Nissl substance, suggestive of cellular injury and early degenerative changes (Figure 4(C)). Due to the differing levels of the thoracic spinal cord between samples, and the variability inherent in Clarke’s column, reliable quantification of neuronal number was not possible.

We counted and assessed lateral horn neurons in three SMA cases and three age-matched controls at birth, 4 months, and 11 months. In sharp contrast to the neuronal loss and cellular degeneration observed in motor and sensory neuronal populations above, we found no evidence of neuronal loss (control = 809.6 ± 64.41 (mean +SEM);SMA = 767.5 ± 77.28 lateral horn neurons per 1-mm length, Pns 0.70), cellular injury or degenerative morphology in these sympathetic neurons (Figure 4(E–G)).

### Gross spinal cord dimensions reflect aberrant development of grey and white matter in SMA

To assess the gross morphology of the spinal cord, we quantified the cross-sectional area of the whole spinal cord, grey and white matter in control and SMA cases, aged between birth and 1 year (*N* = 6). To simulate growth trajectory, we applied simple linear regression, where *s* indicates the steepness and direction of slope, *R*^2^ is the goodness of fit of the linear regression model and *p* value tests the null hypothesis that the overall slope is zero.

**FIGURE 5:**
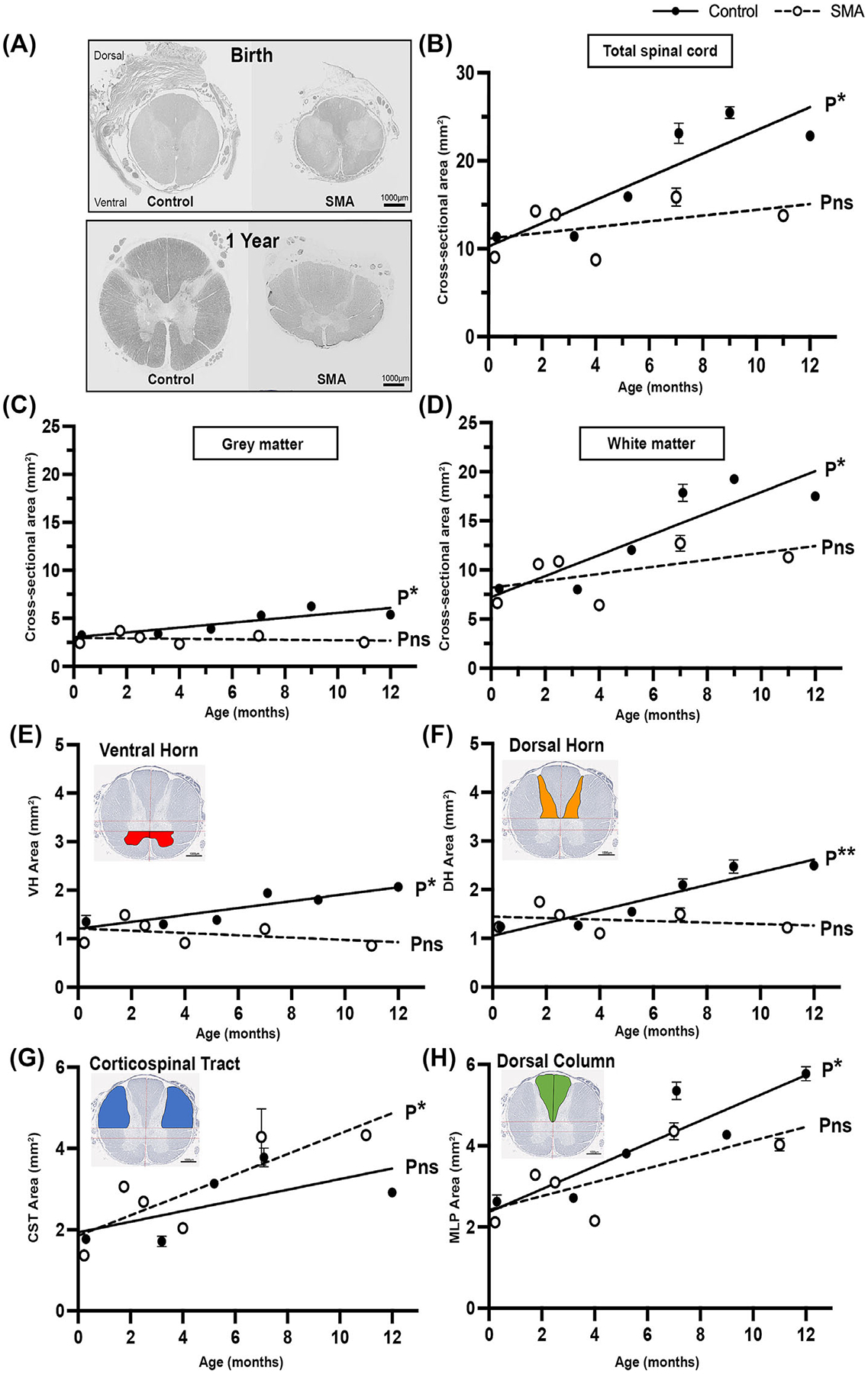
(A) Representative micrographs of control and SMA thoracic spinal cord cross-section at birth and 1 year stained with Sudan black, scale 1000 μm. The cross-sectional area of (B) total thoracic spinal cord, (C) grey matter, and (D) white matter with simple linear regression slope. The cross-sectional area of regions of interest: grey matter (E) ventral horn and (F) dorsal horn, white matter (G) corticospinal tract and (H) dorsal column with simple linear regression slope, control (black) and SMA (white). *N* = 6. *p* value notation illustrates significant elevation of slope from zero, p*, <0.05, Pns = non-significant change in slope.

The total cross-sectional area of the thoracic spinal cord was approximately 40% smaller in SMA cases compared to controls at 12 months (birth, control = 11.34 μm^2^ ± 0.56 and SMA = 9.04 μm^2^ ± 0.09; and 1 year, control = 22.89 μm^2^ ± 0.44 and SMA = 13.80 μm^2^ ± 0.32). Linear regression analyses indicated a significant increase in spinal cord cross-sectional area during the first year of life in control cords (*s* = +1.32, *R*^2^ = 0.77, *F*[1,4] = 13.42, *p = 0.02), while SMA spinal cords showed marginal but non-significant increase in cross-sectional area over the same period (*s* = +0.33, *R*^2^ = 0.19, *F*[1,4] = 0.96, Pns = 0.38) (Figure 5 (A,B)).

During early postnatal life, increasing grey matter should reflect the growth of neuronal cell bodies and the sprouting of dendritic processes [34]. In contrast, but consistent with the substantial motor neuron loss seen, SMA grey matter had not increased in area over the first year (*s* = −0.02, *R*^2^ = 0.03, *F*[1,4] = 0.14, Pns = 0.73), (Figure 5 (C)). White matter also enlarged during the first year of life in control spinal cord, increasing >2-fold (*s* = +1.07, *R*^2^ = 0.77, *F*[1,4] = 13.55, p* 0.02). SMA white matter also increased in the area, but the rate of increase was slower, and as a result remained consistently smaller than in control spinal cord (*s* = +0.36, *R*^2^ = 0.29, *F*[1,4] = 1.64, Pns = 0.27) (Figure 5(D)).

To determine region-specific changes, ventral horn (motor) and dorsal horn (sensory) grey matter, lateral corticospinal tract (descending) and dorsal column (ascending) white matter tracts were assessed in isolation.

**FIGURE 6:**
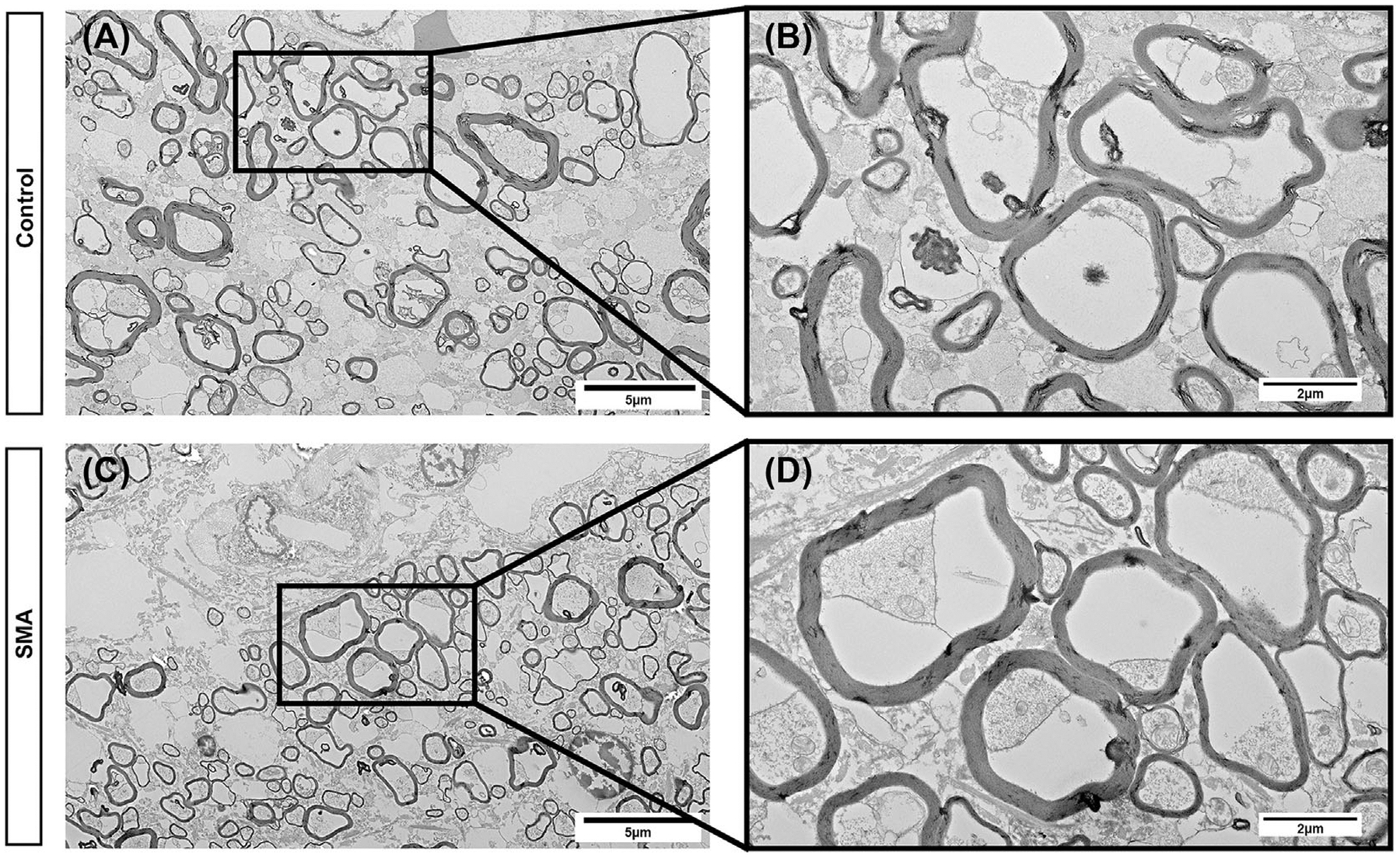
Low magnification representative transmission electron micrographs of myelinated white matter tracts in control (A) and SMA (C) thoracic spinal cord, scales 5μm. High-powered representative transmission electron micrographs of control (B) and SMA (D) myelinated axons showing healthy myelinated axons, with surrounding compact myelin sheaths, scale, 2 μm.

When segregated, grey matter ventral and dorsal horns both significantly increased in absolute area between birth and 1 year in the control spinal cord: ventral horn (*s* = +0.07, *R*^2^ = 0.78, *F*[1,4] = 14.27, p* = 0.02) and dorsal horn (*s* = +0.13, *R*^2^ = 0.89, *F*[1,4] = 31.53, p** = 0.005). In contrast, SMA ventral and dorsal horns were not enlarged, ventral horn (*s* = −0.02, *R*^2^ = 0.14, *F*[1,4] = 0.64, Pns = 0.47) and dorsal horn (*s* = −0.02, *R*^2^ = 0.06, *F*[1,4] = 0.27, Pns = 0.63) (Fig, 5(E,F)). Most surprisingly, the lateral corticospinal tract of SMA spinal cord increased in absolute area (*s* = +0.25, *R*^2^ = 0.70, *F*[1,4] = 9.34, p* = 0.04), and was, in fact, larger than controls after 6 months of age (*s* = +0.13, *R*^2^ = 0.41, *F*[1,4] = 2.10, Pns = 0.24) (Figure 5(G)). The SMA dorsal column did increase in area over increasing survival times (*s* = +0.17, *R*^2^ = 0.53, *F*[1,4] = 4.50, Pns = 0.10) but only to approximately 70% of control area (*s* = +0.28, *R*^2^ = 0.80, *F*[1,4] = 16.06, p* = 0.02) (Figure 6(H)).

SMA spinal cord dorsal and ventral grey matter both failed to increase in absolute area postnatally, irrespective of survival times. Motor and sensory white matter tracts exhibited differential patterns of growth. The increase in the descending corticospinal tract was most pronounced and surprising, especially in comparison to the ascending dorsal column.

### Myelinated axons of the white matter tracts appear normal in SMA

The significant changes in the gross morphology of white matter tracts, lead us to next look for any evidence of axonal changes in the white matter. Regions of white matter were selected at random from SMA and control spinal cord from a subset of individuals, aged between birth and 4 months, and assessed by transmission electron microscopy (TEM). No overt abnormalities of myelinated axons were observed (Figure 6). In both controls and SMA individuals, healthy myelinated axons surrounded by compact myelin sheaths were present (Figure 6(B,D)). We did not observe aberrant development or degenerative morphology within axons or myelin sheaths. These findings suggest that axonal loss or demyelination of ascending or descending spinal cord tracts are not key events at the end of life in SMA.

## DISCUSSION

The spinal cord is the primary location of disease pathology in spinal muscular atrophy (SMA), but our understanding of SMA pathology is based upon few samples and inadequate methodologies. Therefore, what we regard as the foundational neuropathology of SMA patients is questionable. Here, we have applied unbiased quantitative techniques to gather data from a larger group of patients, with similar genotypes and severe disease.

### Neuronal loss in the ventral horn

We show a substantial loss of neurons in the ventral horn, which will include alpha and gamma motor neurons. This equates to a deficit of approximately 94–110,000 ventral horn neurons in severe infantile SMA patients at the end of life. Motor neuron loss in the thoracic spinal cord has not previously been quantified in SMA, but the vulnerability of the thoracic motor pool is known [35]. Respiratory failure remains the leading cause of fatality in SMA Type I and II patients; however, patients also suffer from severe weakness of the trunk resulting in progressive scoliosis and mechanical restriction of the chest wall, further affecting prognosis [19, 21, 36]. Indeed, an early sign of severe infantile SMA is the inability to ‘logroll’: maintain a straight trunk when the pelvis or shoulder girdle is turned, which is facilitated by the intercostal and abdominal wall musculature [25]. In comparison with other spinal segments, previous studies have reported 84%–95% loss of cervical ventral horn neurons, and 78%–95% of lumbar ventral horn neurons; however, different quantitative methods were applied [37–39]. Additionally, emerging phenotypes in treated patients, including scoliosis and consequent reduction in motor ability, indicate that truncal weakness may persist following treatment, possibly as the result of early degeneration of the thoracic motor neurons [26]. Extrapolating our thoracic cord data to the length of the newborn spinal cord (14–16.6 cm [40]), we estimate the total number of ventral horn neurons in the control spinal cord to be approximately 135–160,000, while the SMA spinal cord contains only 41–50,000 neurons at the end of life. We did not assess the levels of SMN protein in surviving neurons, but our previous work has suggested that the levels of SMN vary considerably within both wild-type and SMA neuronal populations and that neurons with the, relatively, highest SMN levels are most resistant to cell stress-induced death and therefore most likely to constitute the remaining neurons [41].

Although the absolute number of neurons does not change after birth, continual growth and elongation of the spinal cord in childhood result in a gradual decrease in neuronal density in the control spinal cord, which is reproduced in SMA cases. At the end of life, SMA cases possess on average 30% of the ventral horn neuron population of controls in the thoracic spinal cord. Considerable neuronal loss was observed in the newborn SMA case, suggesting a significant prenatal loss of neurons. Prolonged or enhanced programmed cell death has been described in a cohort of six SMA foetuses [42, 43]. It is therefore likely that some SMA patients are born with substantial motor neuron loss, which may underlie the observations of ‘non-responders’ reported in clinical trials, and in fatalities after treatment [35, 44, 45].

### Remaining small neurons in the ventral horn

The literature suggests that neuronal cell bodies increase in size as they mature and sprout dendritic processes in healthy spinal cords after birth [34]. However, our data show that control soma diameters did not significantly increase after birth in the thoracic spinal cord. In the SMA ventral horn, surviving neurons were significantly smaller than age-matched controls, with a complete absence of large ventral horn neurons at all survival times (1 week to 12 months).

This absence of large neurons and remaining small neurons have historically been taken as evidence for selective loss of alpha motor neurons in SMA; however, this idea originated from a single study which reported only a lack of all large neurons [39]. Absence cannot be assumed to indicate loss, and assertions of specific loss of alpha motor neurons cannot be made without a time series, which is unattainable in an autopsy study. While data reported here was also gathered from the measurement of cell soma, care was taken to ensure neuronal diameter was measured through only the central profile of the neuron so as not to underestimate neuron size. More widespread neuronal loss may underlie muscle spindle abnormalities in patients with mild SMA, due to a loss of gamma motor neurons in longer-surviving patients [46, 47]. We were unable to differentiate alpha and gamma neurons based on size alone, as even in controls there was no bimodal peak, but rather a continuous distribution of soma size. Identification of gamma motor neurons would require molecular characterisation of alpha from gamma motor neurons, which is not currently possible in human tissue [48].

While it is entirely plausible that large alpha motor neurons are specifically lost in SMA, the lack of all large neurons even in the youngest SMA cases of seven postnatal days would provide a very limited time window to both develop and then lose this population of neurons. Alternatively, the surviving population of small neurons may be either the result of degenerative shrinkage as a prelude to cell death, or a failure to grow normally due to aberrant development and maturation. Small neurons in severe SMA Type I patients alongside similarly small and developmentally immature axons suggest that these may be immature motor units which are actively degenerating and will be lost later in postnatal life [30]. Simic et al. [49] also observed immature, mis-migrated motor neurons in the ventral roots and spinal cord white matter. We did not however observe mis-migrated motor neurons in this cohort of SMA patients but were unable to examine the ventral outflow or ventral roots where these neurons are commonly observed. Unfortunately, from this post-mortem data, it is not possible to determine whether remaining, small neurons are the result of developmental or degenerative processes, but they underpin the reduced grey matter area seen in the SMA spinal cord. Finally, others have suggested that similarly small neurons may represent a developmentally immature population which can be rescued by early therapeutic intervention [30]. Additional work is required to address these key questions.

### Morphology of remaining neurons in the ventral horn

The derivation of disease mechanisms based on the presence of cellular features in remaining neurons in post-mortem tissue can also be misleading. Chromatolysis is a reactive change in a neuronal cell body in response to damage, characterised by reduction or redistribution of Nissl positive organelles, displacement of the nucleus to the periphery and a rounded neuronal cell soma [50]. This morphology reflects cytoskeletal reorganisation and activation of protein synthesis for repair and regeneration, but can also precede neuronal loss in neurodegenerative disease [51]. Although the term chromatolysis is widely used to describe many forms of injured neurons, it strictly refers to the reorganisation of Nissl substance within the cell body, and contrasting literature has led to a debate as to whether central chromatolysis is a suitable hallmark of SMA [16, 38, 42, 49, 52–67]. In SMA spinal cord, we report widespread abnormal phenotypes of the surviving SMA ventral horn neurons. However, only a proportion of these displayed a morphology that can be considered characteristically chromatolytic. Swelling of the cell body or ‘ballooned’ neurons were not observed. We also observed neurons with degenerative phenotypes that cannot be attributed to the chromatolytic morphology, including complete loss of Nissl substance, nuclear disruption and small cells with intense cytoplasmic staining and an uneven periphery. Interestingly, frequently described phenotypes of SMA motor neurons, including peripherally located and less developed Nissl substance, rounded cell soma and fewer neurites, are generally described as features of degenerative chromatolysis, but are also evidence of immaturity [66, 68]. SMA motor neurons have previously been described as shrunken, chromatolytic and pyknotic. These phenotypes areall common features of apoptosis, which is also known to result in cellular shrinkage [38, 47, 55, 57, 69–71]. Chromatolysis is a complex and poorly understood phenomenon, with multiple intrinsic and extrinsic agonists [50, 51, 72, 73] and cannot alone be used to determine disease aetiology.

### Clarke’s column and lateral horn neurons

Features of cellular injury were also observed in neurons of Clarke’s column, a secondary sensory neuronal population, involved in unconscious proprioception of the lower limbs through the dorsal cerebellar circuit [74]. Despite involvement in the somatosensory rather than the motor system, early signs of insult, including mild chromatolysis of neurons, astrocytosis and microglial infiltration, with neuronal loss observed in long-surviving patients are reported [53–62]. Taken together with our findings, these suggest that neurons of Clarke’s column exhibit degenerative changes later in disease progression, and may be secondary to motor neuron loss, caused by interruption to the motor circuit.

In contrast, we found no degenerative changes or neuronal loss in lateral horn sympathetic neurons, involved in control of the autonomic nervous system. Autonomic dysfunction is reported in a small proportion of SMA patients, with symptoms including sympathetic vagal imbalance, blood pressure fluctuations, irregular skin response to temperature changes and adrenergic hyperactivity [75–77]. Degeneration of lateral horn neurons is common in conditions which cause progressive autonomic dysfunction; however, no association has been determined between lateral horn neuron loss and severity of autonomic dysfunction, suggesting the importance of the sympathetic ganglia and dorsal vagal nuclei on symptomatic presentation [78, 79]. Involvement of other components of the autonomic pathway may then account for the autonomic dysfunction observed in some SMA patients.

### Macroscopic spinal cord anatomy

Normal, healthy spinal cord increases in all dimensions in postnatal life, predominately due to axonal growth, extension and myelination [80]. Small spinal cords observed in SMA cases reflected differences in both grey and white matter. SMA grey matter did not increase in area, irrespective of survival times, and both ventral and dorsal horns failed to attain normal postnatal size, suggesting size differences cannot simply be the result of motor neuron loss. The ascending dorsal column white matter tract also failed to achieve normal control dimensions, while the lateral corticospinal tract was larger than in controls. In an MRI study of adult patients, anterior–posterior atrophy of the lower cervical cord showed up to 23% reduction in width, which may suggest the SMA spinal cord never achieves normal dimensions [81]. Further investigation of the corticospinal tract would be required to confirm hypertrophic changes; however, enlargement may reflect compensatory hypertrophy of the upper motor circuitry in an attempt to overcome lower dysfunction. Axonal remodelling and corticospinal tract enlargement through additional, larger axonal projections are observed in animal models of stroke, allowing motor map reorganisation for attempted recovery of motor function [82].

The morphology of myelinated axons did not exhibit any overt phenotypes of deficient myelination or myelin degeneration; therefore, differences observed in overall areas may reflect changes in axonal number or calibre, but which we were not able to ascertain. Aberrant central white matter tracts in SMA are commonly described as myelin pallor, specifically of the dorsal column, spinocerebellar tract and anterior and lateral corticospinal tracts [38, 47, 57, 62, 64, 83, 84]. Due to the small sample size, lack of age-matched controls and randomisation of EM blocks, we were unable to assess individual white matter tracts of the SMA spinal cord at aged-matched time-points in development. During the first year of life, the spinal cord is dynamic, with maturation and myelination of specific white matter tracts occurring at different times. To determine the reason for white matter abnormalities in the SMA spinal cord, further studies should seek to quantify axonal density and calibre in carefully aged-matched spinal cords.

## CONCLUSION

Our findings demonstrate that while motor neuron loss is a significant feature of SMA, pathology is not limited to the ventral horn. In addition, long-accepted dogmas of SMA disease pathology, including selective loss of alpha motor neurons, are based upon weak evidence and should be challenged. It is time to acknowledge the breadth of pathology which occurs in response to low levels of SMN protein in SMA. These data provide a new normal upon which to base future research to further appreciate significant pathologies beyond the loss of alpha motor neurons in the spinal cord, evaluate the success of current therapies, and aid in the design of novel ones.

## Figures and Tables

**TABLE 1:** Patient demographics. Age of death and age of onset of SMA symptoms (days [d]/months [m]), sex, cause of death. *SMN2* copy number, post-mortem interval (PMI), if known, and application of light microscopy (LM) or transmission electron microscopy (TEM).

	Age of death (days/months)	Age of SMA onset (days/months)	Sex	Cause of death	SMN copy number (*SMN1/SMN2*)	PMI (h)	Application
SMA Patients	7 d	Unknown	Female	SMA Type 0	Unknown	Unknown	LM
	1.75 m	0.5 m	Male	SMA Type I	0/2	7	LM
							TEM
	2.5 m	1 m	Female	SMA Type I	0/2	7	LM
							TEM
	4 m	4 m	Male	SMA Type I	0/2	4	LM
	7 m	0.75 m	Female	SMA Type I	0/2	25	LM
							TEM
	11 m	5 m	Male	SMA Type I	0/2	32	LM
	11 m	Unknown	Female	SMA Type I	0/2	Unknown	TEM
Control Samples	1d	-	Unknown	Unknown	-	26	TEM
	4d	-	Unknown	Unknown	-	69	TEM
	9 d	-	Female	Unknown	-	Unknown	LM
	3.2 m	-	Male	Sudden unexplained infant death	-	27	LM
	5.2 m	-	Female	Viral syndrome/focal acute pneumonia	-	31	LM
	7.1 m	-	Male	Asphyxia	-	24	LM
	9 m	-	Male	Pneumonia	-	14	LM
	12 m	-	Male	Sudden infant death syndrome	-	27	LM

**TABLE 2:** Absolute number of ventral horn neurons and motor milestones. Stereological quantification of the absolute number of ventral horn neurons in 1-mm length of the thoracic spinal cord, of SMA patients and similar age-matched control individuals, and the highest motor milestone achieved by each.

Group	Age	Absolute number of VH neurons (per 1-mm length thoracic cord)	Motor milestones achieved
SMA	7 days	525	Unknown
Control	9 days	1000	Supine head control and limb movement
SMA	1.75 months	413	No head control, no supine to prone roll
SMA	2.5 months	188	No head control, no supine to prone roll
Control	3.2 months	1088	Steady head control and supine to prone roll
SMA	4 months	325	No head control, no supine to prone roll
Control	5.2 months	888	Steady head control and supine to prone roll
SMA	7 months	188	No head control, limited supine to prone roll
Control	7.1 months	1100	Maintenance of seated position with unsupported head control
Control	9 months	863	Pulls to stand and takes unassisted steps
SMA	11 months	150	Seated head control and limited roll
Control	12 months	850	Takes unassisted steps, with improvement to independent walking

Abbreviation: SMA, spinal muscular atrophy.

## Data Availability

The data that support the findings of this study are available on request from the corresponding author. The data are not publicly available due to privacy or ethical restrictions.

## Key Points

- Thoracic motor neuron loss is key in spinal muscular atrophy prognosis but has not previously been assessed.
- Thoracic ventral horn neuron loss is substantial, even in patients with short postnatal survival.
- The morphology of the remaining ventral horn neurons suggests significant cellular damage.
- Clarke’s column neuronal morphology indicated cellular injury; however, lateral horn neurons were preserved.
- Postnatal spinal cord growth was stunted, reflecting changes in both grey and white matter.
